# Relationship of Vitamin D Deficiency with Cervical Vertebral Maturation and Dental Age in Adolescents: A Cross-Sectional Study

**DOI:** 10.1155/2022/7762873

**Published:** 2022-11-22

**Authors:** Golnoosh Azarbakhsh, Parastoo Iranparvar, Azita Tehranchi, Mahkameh Moshfeghi

**Affiliations:** ^1^School of Dentistry, Shahid Beheshti University of Medical Sciences, Tehran, Iran; ^2^Athena Institute, Faculty of Science, Vrije Universiteit, Amsterdam, Netherlands; ^3^Department of Pediatric Dentistry, School of Dentistry, Shahid Beheshti University of Medical Sciences, Tehran, Iran; ^4^Dentofacial Deformities Research Center, Research Institute of Dental Sciences, School of Dentistry, Shahid Beheshti University of Medical Sciences, Tehran, Iran; ^5^Department of Oral & Maxillofacial Radiology, School of Dentistry, Shahid Beheshti University of Medical Sciences, Tehran, Iran

## Abstract

**Background:**

Considering the high prevalence of vitamin D deficiency and its effect on growth and development, the assessment of the dental age and skeletal age with regard to vitamin D deficiency status could influence the treatment planning of growth modification treatment. This study aimed to assess the relationship between vitamin D deficiency, cervical vertebral maturation (CVM) as an indicator of skeletal age, and dental age in adolescent patients.

**Methods:**

In this cross-sectional study, the chronological age of 52 orthodontic patients aged between 10 and 14 years was recorded, and their serum level of vitamin D was determined using a radioimmunoassay test. The patients were then divided into three groups based on their serum vitamin D level: severe deficiency, moderate deficiency, and the control group with normal vitamin D. The panoramic radiographs of patients were assessed to determine their dental age using Demirjian's method. CVM was determined on lateral cephalograms using Baccetti's classification to specify the skeletal age. Data were analyzed using a *t*-test, linear regression, ordinal logistic regression, and Pearson's correlation coefficient (at *P* < 0.05, confidence interval = 95%).

**Results:**

Skeletal age showed a significant difference between the group with severe vitamin D deficiency and the control group (*P*=0.01); however, such difference was not observed between the group with moderate vitamin D deficiency and the control group (*P*=0.12). Dental age was not significantly different between the groups with vitamin D deficiency and the control group (*P*=0.26 for severe, and *P*=0.39 for moderate deficiency).

**Conclusions:**

A less advanced skeletal maturation was observed in adolescents with severe vitamin D deficiency; however, dental development was not affected by this deficiency. Vitamin D status is better to be considered in decision-making for the initiation of growth modification orthodontic treatments.

## 1. Introduction

Vitamin D plays a fundamental role in bone development and muscle function. It eliminates calcium and phosphate from the kidneys, suppresses the parathormone, and induces the osteoblasts to form new bone. Vitamin D deficiency causes osteomalacia in children, results in recurrence and aggravation of osteopenia and osteoporosis, and can lead to bone fracture in adults. It can also cause growth retardation, muscle weakness, skeletal deformities, and hypocalcemia [1]. The role of vitamin D deficiency in the etiology of other disorders such as diabetes mellitus, multiple sclerosis, and auto-immune problems has also been established [2].

Vitamin D deficiency has a high prevalence in children and adults. Evidence shows that in the past two decades, vitamin D deficiency has become increasingly prevalent in China, Turkey, India, Iran, and Saudi Arabia with a prevalence rate ranging from 30% to 93% [3, 4]. The prevalence of vitamin D deficiency is higher in countries with insufficient exposure to sunlight, insufficient dietary intake of vitamin D, and air pollution [5]. Vitamin D deficiency is now a pandemic, and the main reason is believed to be insufficient exposure to sunlight [1].

Data obtained from the growth charts of children can help dental clinicians and orthodontists find the best time for the initiation of growth modification treatments [6]. It also helps in estimating children's growth and development status to prevent oral and dental disorders [7]. Many physical parameters such as height, weight, skeletal maturity, and dental development can be used to determine the growth and development stage [8]. Dental age is determined based on the order of tooth eruption, while skeletal age is determined by assessing the maturation of the C2 to C4 cervical vertebra. Cervical Vertebral Maturation (CVM) is commonly used to determine skeletal age [9].

A delay in dental age and skeletal age, when compared to the chronological age, has been observed in patients with a growth hormone deficiency, HIV-positive patients, those with Celiac disease, and underweight patients [10–13]. On the other hand, accelerated dental and skeletal age has been reported in obese children [14, 15]. Patients with vitamin D deficiency may also experience retardation in their growth and development [16].

Considering the high prevalence of vitamin D deficiency, its effect on growth and development, and the importance of determining patients' growth status in the treatment planning of growth modification treatments, the need for assessment of the dental age and skeletal age with regard to vitamin D deficiency status is highlighted. This study aimed to assess the association of vitamin D deficiency with dental and skeletal age in 10 to 14-year-old adolescents, using panoramic radiographs and lateral cephalograms.

## 2. Methods

This cross-sectional study evaluated 52 orthodontic patients aged between 10 and 14 years presenting to the Orthodontic Department at Shahid Beheshti School of Dentistry, Tehran, Iran, following the appropriate institutional ethics approval (IR.SBMU.RIDS.REC.1394.11). The study design was in accordance with the Helsinki Declaration of Human Rights. All the patients had panoramic radiographs and lateral cephalograms obtained for their orthodontic treatment, therefore eliminating the need for unnecessary radiation exposure during the research. All the patients and their parents were informed about the study and the parents signed the informed consent forms.

The sample size was calculated to be 51 (*N* = 17 in each group) using Power and Sample Size Calculation software version 3.0.43. The inclusion criteria consisted of an age between 10 and 14 years, having panoramic radiographs and lateral cephalograms taken for orthodontic treatment, willingness to participate in the study, and presence of signed informed consent forms by the parents.

The exclusion criteria were sunbathing more than once a week, taking vitamin D supplements, consumption of vitamin D-rich foods and drinks more often than once a day since the initiation of orthodontic treatment, a history of systemic diseases, medication intake, craniofacial syndromes, the bilateral missing of mandibular teeth, poor quality of radiographs, and not performing blood test in the designated laboratory. It should be mentioned that the exclusion criteria were evaluated according to the parent's responses to the questions.

After obtaining the parents' consent, all patients underwent blood tests in the designated laboratory to determine their serum 25-hydroxy vitamin D level, using chemiluminescence immunoassays (CLIA). All blood tests were performed during the fall season to eliminate the effect of seasonal changes in vitamin D levels. The patients and their parents were informed of the test results and were educated about the role of vitamin D in the body. The test results were provided to the parents, and they were referred for medical consultation if needed. The patients were classified into three groups according to their 25-hydroxy vitamin D (25(OH)D) serum level.

  Normal: 25(OH)D > 30 ng/mL; sufficient vitamin D
  Low: 15 < 25(OH)D ≤ 30 ng/mL; moderate vitamin D deficiency
  Very low: 25(OH)D ≤ 15 ng/mL; severe vitamin D deficiency

**Figure 1:**
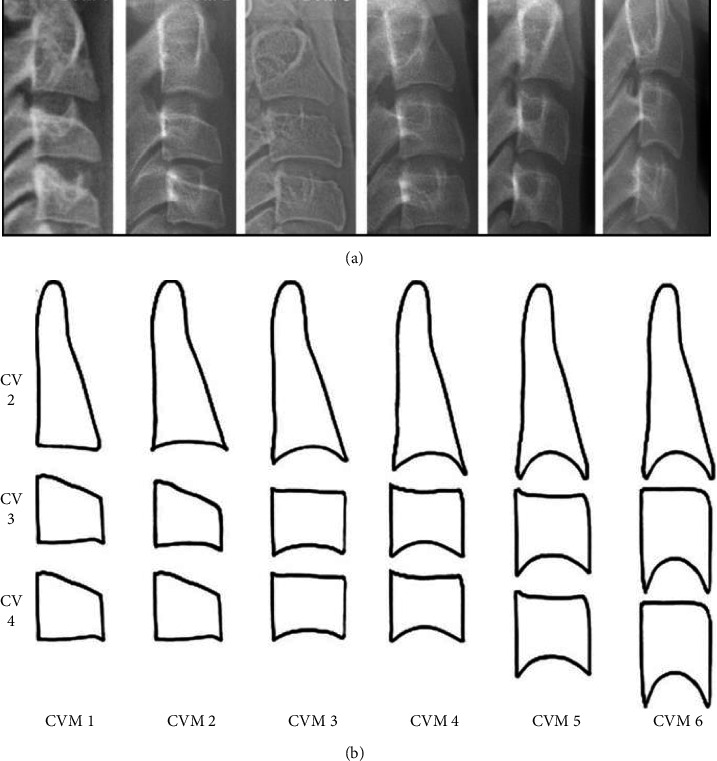
Bacceti's method of determining cervical vertebral maturation stages for estimation of skeletal age. CVM 1: the inferior border of C2–C4 does not have any concavities. The body of C3 and C4 is trapezoidal (the superior border of cervical vertebrae has a slope from the posterior towards the anterior). Maximum mandibular growth occurs around two years after this stage. CVM 2: concavity exists in the inferior border of C2. The body of C3 and C4 is still trapezoidal. Maximum mandibular growth occurs approximately one year after this stage. CVM 3: concavity exists in the inferior border of C2 and C3. The body of C3 and C4 may be trapezoidal or horizontally rectangular. Maximum mandibular growth occurs in less than one year after this stage. CVM 4: concavity exists in the inferior border of C2, C3, and C4. The body of C3 and C4 is horizontally rectangular. Maximum mandibular growth has occurred one to two years prior to this stage. CVM 5: concavity still exists in the inferior border of C2, C3, and C4. The body of at least one of the C3 and C4 is square-shaped. If not, it is horizontally rectangular. Maximum mandibular growth has ended at least one year prior to this stage. CVM 6: concavity still exists in the inferior border of C2, C3, and C4. The body of at least one of the C3 and C4 is vertically rectangular. If not, it is square-shaped. Maximum mandibular growth has ended at least two years prior to this stage.

To determine the skeletal age of patients, CVM stage analysis of the second, third and fourth cervical vertebrae (CV2-CV4) on lateral cephalograms (Soredex® Cranex-D, Tuusula, Finland) was performed according to Baccetti's modified protocol [17]. The radiographs were converted to JPEG (Joint Photographic Experts Group) file format using Digora® software version 8.2 (Strasbourg, France) and observed using Photoshop software (Adobe®, San Jose, CA, USA). Magnification of ×150 was used for more accurate observation when required. In order to prevent errors in the determination of skeletal age, the evaluation of all lateral cephalograms was confined to the cervical vertebrae by the observer to determine the skeletal age without observing the teeth. Two calibrated, trained orthodontists, blinded to the chronological age and serum vitamin D level of patients, independently determined the CVM stage and the skeletal age. In cases of disagreement, a third blind observer (orthodontist) also reviewed the radiographs. For each patient, two variables, including the presence/absence of concavity in the inferior border of CV2, CV3, and CV4; and the body shape of CV3 and CV4 (trapezoidal, horizontal rectangular, square, and vertical rectangular) were evaluated, as presented by Baccetti [17]. According to these two variables, the patients were assigned to one of the skeletal maturation stages (CVM1 to CVM6) as presented in Figure 1.

**Figure 2:**
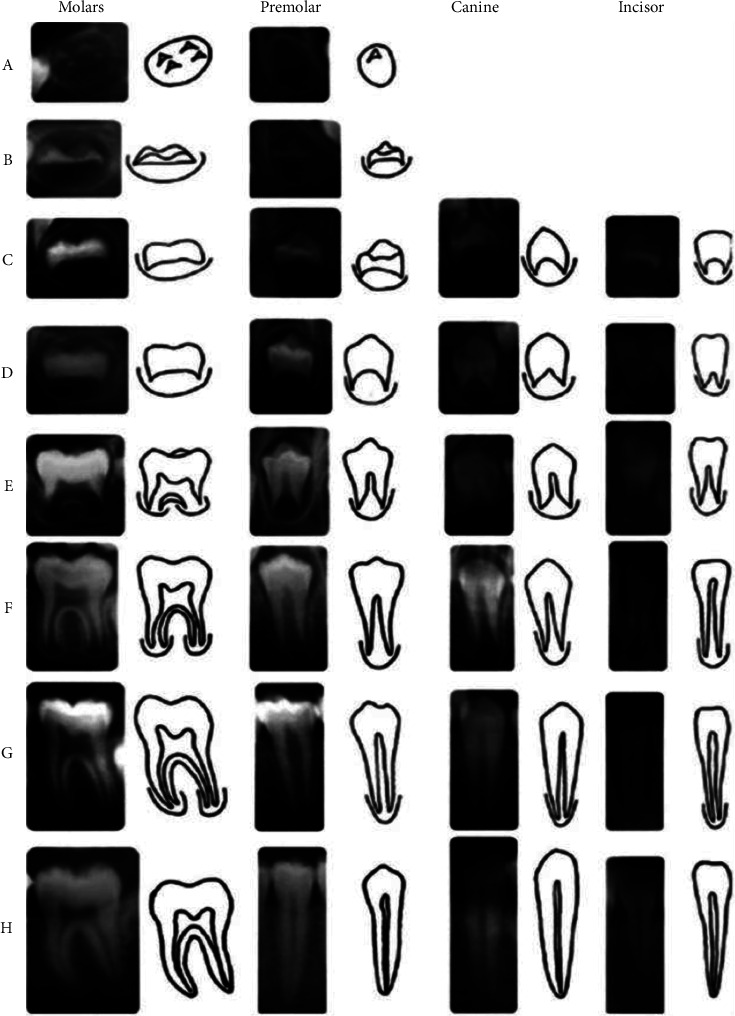
Demirjian's method of determining dental growth stages for estimation of dental age.

The panoramic radiographs (Soredex® Cranex-D, Tuusula, Finland) were also independently evaluated by two calibrated orthodontists, blinded to the chronological age and serum vitamin D level of patients, to determine the dental age according to Demirjian's method [18]. For this purpose, the calcification stage of seven permanent teeth in the mandibular left quadrant (from tooth number 24 to 18) was scored individually from A to H. The standard scores of teeth were separately reported for males and females. A total score of dental maturation, which was the sum of individual scores, was reported for each individual. This total score was converted to dental age (in years) according to a standard table. The scores given to different calcification stages of teeth were determined according to Demirjian and Goldstein, as presented in Figure 2 [18].

Data were analyzed using SPSS (Statistical Package for the Social Sciences) software version 21.0 (SPSS® Inc., IL, USA). After the primary assessment of all radiographs, 10 radiographs were randomly selected and re-evaluated after two weeks to assess the intraobserver error. The kappa coefficient was also calculated to assess the interobserver agreement. The mean kappa coefficient for the intraobserver agreement was also calculated for dental age and skeletal age determination.

The mean dental age of patients was compared among the groups using a *t*-test. The linear regression model was applied to compare dental age among the three groups after controlling for chronological age and gender. Since the CVM stage was a descriptive variable, an ordinal logistic regression model was used to compare the skeletal age among the groups after controlling the chronological age and gender. Pearson's correlation test was used to assess the correlation between dental age and chronological age in each group. *P* < 0.05 was considered statistically significant.

## 3. Results

A total number of 52 participants were included in the present study. The intraclass correlation coefficient was found to be excellent for both the calcification stage of teeth (0.99) and the CVM stage (0.95). The mean kappa coefficient for the intraobserver agreement was 95% for the determination of skeletal age and 99.9% for the determination of dental age. The mean kappa coefficient calculated to assess the interobserver agreement was found to be 92.6% for the determination of CVM stage, and 99.9% for the determination of dental age, which indicated excellent agreement between the two observers. This value was 100% for the determination of maturation stages for the central incisor and first molar teeth.

Evaluation of gender distribution among the three groups revealed no significant difference in the distribution of males and females (*P*=0.94). Similarly, the distribution of chronological age between the groups was not significantly different (*P*=0.29).

After controlling for chronological age and level of vitamin D, the results showed that dental age and skeletal age were not significantly different between males and females (*P* > 0.05).

According to the results of Pearson's correlation coefficient, a positive correlation was detected between dental age and chronological age in all patients (*r* = 0.6, *P* value <0.001). A positive correlation between dental age and skeletal age was also established (*r* = 0.7, *P* value <0.001, according to Spearman's rank correlation).

Table 1 presents the distribution of CVM stages among the three groups based on vitamin D levels. The highest frequency of CVM 1, CVM 2, and CVM 3 was noted in the severe vitamin D deficiency group, while the highest frequency of CVM 4 and CVM 5 was noted in the control group with sufficient serum levels of vitamin D. Since all patients were between 10 and 14 years old, none of them was in CVM stage 6.  Table 2 shows the mean ± SD of dental age among the three groups based on vitamin D levels. As presented in Table 3, after controlling for chronological age and gender, the results revealed that dental age was not significantly different among the three groups (*P* > 0.05). However, a comparison of skeletal age among the three groups revealed a significant difference between the group with severe vitamin D deficiency and the control group (*P*=0.01). The same significant difference was not observed between moderate vitamin D deficiency and control (*P*=0.12).

## 4. Discussion

The developmental stage of adolescent orthodontic patients has a significant role in treatment timing and can be determined using different indicators. Presently, the dental age and skeletal development stage are used as the main indices of skeletal growth and maturation in dental practice [19]. These two variables are more valuable in clinical assessments than chronological age and gender-related characteristics [11]. Vitamin D plays a fundamental role in the development, metabolism, and function of hard tissues; therefore, its deficiency contributes to growth retardation [1].

This study assessed the association of vitamin D deficiency with skeletal age and dental age in adolescents compared to a normal control group. The results indicated that, although there was no significant difference in dental age between the group with vitamin D deficiency and the control group, the skeletal growth delay might be attributed to severe vitamin D deficiency [20]. This finding could help dentists to better arrange orthodontic treatment plans according to the patient's skeletal age while considering their serum level of vitamin D. The absence of correlation between vitamin D level and dental development in adolescents could be attributed to the timing of major dental development, happening earlier in life.

In the present study, skeletal age was determined based on lateral cephalogram using the method described by Baccetti et al. [17]. Evidence shows a highly significant correlation between this method and the skeletal age determined based on hand-wrist radiography using Bjork's method in both males and females. Therefore, the determination of the CVM stage on lateral cephalograms is the most favorable method for the assessment of skeletal age in orthodontic patients, since it is faster and has a lower patient radiation dose [21, 22].

Several techniques are available to estimate and determine dental age; Demirjian and Goldstein method [18] is one of the most used methods for the assessment of dental age [23]. However, the selection of dental age assessment methods depends on the study population [18, 24, 25]. In the present study, considering the accuracy, applicability, and reproducibility of Demirjian's method for assessment of dental age in both males and females, this method was employed [26, 27].

In the present study, a positive correlation between dental age and chronological age and also a positive correlation between dental age and skeletal age was established in all patients irrespective of their vitamin D level. Cisternas et al. in their study on 657 panoramic radiographs and lateral cephalograms of 5 to 15-year-olds found a significant correlation between dental and skeletal maturation, which is in line with the results of the present study [28]. Similarly, Howell in his study on 90 panoramic radiographs and lateral cephalograms, showed a significant correlation between dental age determined by Demirjian's method and skeletal maturation determined by Baccetti's method. He recommended the assessment of panoramic radiographs for the calculation of dental age using Demirjian's method as a tool for the estimation of skeletal maturation state, which could be helpful in orthodontic treatment planning for British patients [29].

The effect of vitamin D on dental maturation has been evaluated in a study by Backstrom et al., indicating that vitamin D supplementation in immature infants did not significantly enhance dental maturation. However, the consumption of vitamin D supplements by pregnant mothers would improve dental maturation in their infants [30]. Although the present study was performed on adolescents, dental maturation was not observed to be affected by serum vitamin D levels.

Measurement of the serum level of 25-hydroxy vitamin D is the best indicator of vitamin D sufficiency in an individual [31]. However, it should be noted that the serum level of vitamin D is affected by seasonal changes, with the highest and lowest levels in summer and fall, respectively. Therefore, in the present study serum vitamin D level of all patients was measured in the fall season [1, 32]. Chronological age can also affect the serum level of vitamin D [33]. Therefore, studies on serum levels of vitamin D should assess patients in a specific age group. In this study, patients aged between 10 and 14 years were evaluated since most interceptive orthodontic and pediatric dentistry procedures are performed in this age range. Furthermore, the determination of CVM stage and dental age on radiographs has the highest accuracy in this age range [34]. After the age of 14, most teeth have already developed, hence, the accuracy of radiographic assessment would decrease [25, 35].

Considering the association between severe vitamin D deficiency and less advanced skeletal maturation in adolescents aged between 10 and 14 years in the present study, it is important to monitor the serum level of vitamin D in this age group.

One limitation of the present study was associated with convincing the participants for performing blood tests in the designated laboratory and the fluctuations in vitamin D levels depending on the season and for individuals over time. Furthermore, some radiographs in patients' electronic files did not fulfill image quality requirements for the determination of dental or skeletal age. Another limitation was attributed to the fact that some children had received vitamin D supplements as part of their school programs.

Further studies with a larger sample size are required for more definitive conclusions. In addition, longitudinal studies are suggested following the administration of vitamin D supplementation for children with vitamin D deficiency to better evaluate the correlation.

## 5. Conclusions

Within the limitations of the present study, it can be concluded that vitamin D status is better to be considered in decision-making for the initiation of growth modification orthodontic treatments.

## Figures and Tables

**Table 1:** CVM stages by vitamin D (25(OH)D) level category among the study groups.

	CVM stage					Total (%)
	CVM 1 (%)	CVM 2 (%)	CVM 3 (%)	CVM 4 (%)	CVM 5 (%)	
Severe vitamin D deficiency	17.6	47.1	23.5	5.9	5.9	100
Moderate vitamin D deficiency	16.7	27.8	16.7	22.2	16.7	100
Sufficient vitamin D	5.9	17.6	17.6	35.3	23.5	100

Abbreviations used in this table: CVM = cervical vertebral maturation.

**Table 2:** Mean ± SD of dental age among the three study groups based on vitamin D (25(OH)D) levels.

Vitamin D level category	*N*	Mean (SD) of dental age
Severe vitamin D deficiency	17	10.9 (2.2)
Moderate vitamin D deficiency	18	11.6 (2.6)
Sufficient vitamin D	17	12.2 (2.4)
Total	52	11.6 (2.4)

Abbreviations used in this table: SD = standard deviation.

**Table 3:** *P* value^*∗*^ measurements for paired comparisons of dental age and skeletal age among the study groups.

	Dental age	Skeletal age
Severe vitamin D deficiency vs. control (sufficient vitamin D)	0.26	0.01^*∗*^
Moderate vitamin D deficiency vs. control (sufficient vitamin D)	0.39	0.12

^*∗*^Significance level = 0.05, at a confidence interval of 95%, according to linear regression and ordinal regression tests.

## Data Availability

The datasets used and/or analyzed during the current study are available from the corresponding author upon reasonable request.

## Abbreviations

CVM: Cervical vertebral maturation.
